# Combining NDVI and Bacterial Blight Score to Predict Grain Yield in Field Pea

**DOI:** 10.3389/fpls.2022.923381

**Published:** 2022-06-28

**Authors:** Huanhuan Zhao, Babu R. Pandey, Majid Khansefid, Hossein V. Khahrood, Shimna Sudheesh, Sameer Joshi, Surya Kant, Sukhjiwan Kaur, Garry M. Rosewarne

**Affiliations:** ^1^Agriculture Victoria, AgriBio, Centre for Agri Bioscience, Bundoora, VIC, Australia; ^2^Agriculture Victoria, Grains Innovation Park, Horsham, VIC, Australia; ^3^School of Applied Systems Biology, La Trobe University, Bundoora, VIC, Australia; ^4^Centre for Agricultural Innovation, The University of Melbourne, Melbourne, VIC, Australia

**Keywords:** genomic prediction, multivariate model, field pea, grain yield, NDVI, bacteria blight

## Abstract

Field pea is the most commonly grown temperate pulse crop, with close to 15 million tons produced globally in 2020. Varieties improved through breeding are important to ensure ongoing improvements in yield and disease resistance. Genomic selection (GS) is a modern breeding approach that could substantially improve the rate of genetic gain for grain yield, and its deployment depends on the prediction accuracy (PA) that can be achieved. In our study, four yield trials representing breeding lines' advancement stages of the breeding program (S0, S1, S2, and S3) were assessed with grain yield, aerial high-throughput phenotyping (normalized difference vegetation index, NDVI), and bacterial blight disease scores (BBSC). Low-to-moderate broad-sense heritability (0.31–0.71) and narrow-sense heritability (0.13–0.71) were observed, as the estimated additive and non-additive genetic components for the three traits varied with the different models fitted. The genetic correlations among the three traits were high, particularly in the S0–S2 stages. NDVI and BBSC were combined to investigate the PA for grain yield by univariate and multivariate GS models, and multivariate models showed higher PA than univariate models in both cross-validation and forward prediction methods. A 6–50% improvement in PA was achieved when multivariate models were deployed. The highest PA was indicated in the forward prediction scenario when the training population consisted of early generation breeding stages with the multivariate models. Both NDVI and BBSC are commonly used traits that could be measured in the early growth stage; however, our study suggested that NDVI is a more useful trait to predict grain yield with high accuracy in the field pea breeding program, especially in diseased trials, through its incorporation into multivariate models.

## Introduction

Field pea or dry pea (*Pisum sativum* L.) is an annual pulse crop widely grown in all major cropping regions, including North America, Russia, India, Australia, etc. (Zohary, 1999), with global production of 15 million tons in FAOSTAT (2020). It is an important staple legume with high nutritional value and high protein content, as well as being able to be harvested as hay or used for forage in poor seasons (Burstin et al., 2011; Amarakoon et al., 2012). In low-input farming systems, field pea is grown as a significant rotational crop, providing benefits of improved soil fertility, a disease break, and giving better options for weed control (Pritchard, 2015; Powers and Thavarajah, 2019). More recently, field pea has been used as a protein source for meat substitution products in the food manufacturing industry due to its low price and lower allergenic nature (Bashi et al., 2019).

Field pea is a cool-season crop, and grain yield can vary dramatically, from 0.5–1 to 4–5 t/ha depending on environmental conditions (Smýkal et al., 2012). Conventional breeding has improved the field pea grain yield by improving desirable traits, such as early vigor, flowering time, and plant type, and by deploying diverse genetic resources (Lejeune-Henaut et al., 2008; Sadras et al., 2012; Singh and Srivastava, 2015; Coyne et al., 2020). Molecular markers have been identified in gene or QTL mapping and association studies for important agronomical traits, although such studies are limited compared with cereal crops (Cheng et al., 2015; Tayeh et al., 2015a; Kreplak et al., 2019). Marker-assisted breeding approaches that have the potential to improve productivity have been outlined (Tayeh et al., 2015b; Pandey et al., 2021).

Diseases are the major constraint to yield in all crop species, and breeding for resistance is a valuable aim. In field pea, grain yield has been shown to be heavily impacted by biotic stresses, such as rust and Ascochyta blight (Bretag et al., 2006; Rai et al., 2011). In recent years, bacterial blight (*Pseudomonas syringae* pv. *pisi* Sackett and *Pseudomonas syringae* pv. *syringae* van Hall ) has become one of the most important diseases in Australia's field pea production (Hollaway et al., 2007). If initiated in the early growth stages, it can cause up to 60% yield loss of susceptible varieties under conducive conditions (Hollaway et al., 2007).

Field-based assessments of disease and yield in variety trials are often challenging due to the subjective nature of measurements. Remote sensing technologies have the potential to objectively assess traits in the field. Sensors deployed in an unmanned aerial system (UAS) have the capacity to become high-throughput platforms (HTP) that can assess many thousands of lines in a short period of time (Huang et al., 2020). Normalized difference vegetation index (NDVI) generated from a high-throughput phenotyping platform has been used to study its association with disease resistance in chickpea (Zhang et al., 2019), plant height in maize (Han et al., 2019), and phenology in rice (Yang et al., 2017). In field pea, NDVI has been used in the study of lodging (Quiros Vargas et al., 2019), and high correlation of around 0.83 was observed between NDVI and yield (Zhang et al., 2021).

Genomic selection (GS) is a modern breeding method that combines genotypes and phenotypes of a training population to predict breeding values in genotyped but not phenotyped individuals by using appropriate statistical models. The breeding values can then be used for selection in the breeding process (Meuwissen et al., 2001). It is assumed that causal variants underlying a trait are in linkage disequilibrium (LD) with at least one marker when using high-density genome-wide molecular markers. Therefore, GS could account for all causal variants and, theoretically, is expected to outperform pedigree or marker-assisted selection (Goddard and Hayes, 2007). GS has been widely studied in cereal crops (Crossa et al., 2017; Robertsen et al., 2019). It has been tested in a diverse pea collection, and high prediction accuracy (PA) was achieved for thousand seed weights (Burstin et al., 2015). Using the recombinant inbred line (RIL) populations, PA for grain yield ranged from 0.19 to 0.30 for cross-validation between populations and within populations (Annicchiarico et al., 2019).

The univariate or single-trait genomic prediction model is the most common method used in crop GS (de Los Campos et al., 2013; Wang et al., 2018; Zhao et al., 2021). However, additional information in genetically correlated traits can be exploited to improve prediction accuracies *via* multivariate models. Such approaches have been investigated in wheat, rice, and soybean (Jia and Jannink, 2012; Bao et al., 2015; Wang et al., 2017; Gill et al., 2021).

In Australia, the field pea breeding program started in the 1970s (Hawthorne et al., 2003), and its production currently averages about 250,000 tons per year, equating to 10–15% of the annual Australian pulse crops. The growing area of field pea covers Victoria, South Australia, Western Australia, and New South Wales (GRDC, 2018). As an important cash crop, incorporating GS to improve grain yield in the future field pea breeding program is critical. The aims of this study were to (1) estimate variance components and heritability for yield, NDVI, and bacterial blight scores (BBSC); (2) calculate the genetic correlation among the three traits; and (3) assess the genomic PA for grain yield by combining NDVI and BBSC with the multivariate models under cross-validation and forward independent prediction scenarios. This study was established to evaluate the potential to predict grain yield for field pea using phenotypic traits collected through HTP field phenotyping and disease scores, and to facilitate the implementation of GS in the field pea breeding program.

## Materials and Methods

### Plant Material and Field Trials

In 2018, the field pea breeding program had four breeding stages, represented as stages 0 (S0), 1 (S1), 2 (S2), and 3 (S3), which had decreasing numbers of entries due to the artificial selection conducted in the breeding cycle. Breeding lines in each cycle were selected from the respective previous staged breeding lines from 2017, i.e., S3 lines were selected from the 2017 S2 lines. The S0 stage consisted of 960 newly generated lines, with S1, S2, and S3 trials having 420, 240, and 144 breeding lines, respectively. Pedigree of all breeding lines are included in the Supplementary Table 1.

All trials were sown in Horsham, Victoria (36°43′58.79″S, 142°05′15.02″E) in a completely randomized block design with two replicates, apart from the S0 trial which was partially replicated. The plots in all trials were arranged in 12 columns, with each plot being 5 m long and 1.25 m wide with five rows spaced at 0.25 m each. Visual scores of BBSC were recorded on a 1–10 scale from each plot when disease infection was at its peak (pod filling stage). Briefly, score 1 represented a plot that had 0–10% plants affected by the disease, 2 represented a plot with 10–20% plants affected by the disease, and so on. Plots were machine-harvested, and grain weights were recorded. The plot weights were converted to yield (tons/ha) using a plot width of 1.75 m. Weather data during the growing season were obtained from a weather station installed at the site (Supplementary Figure 1). Chemical applications were according to local farmer practice.

### Aerial Image Acquisition, Processing, and Analysis

The aerial images were acquired when disease infection was at maximum, at the early pod filling stage, using a multispectral Parrot Sequoia camera (Parrot S. A., Paris, France) attached to a UAS. The camera had four discrete channels, namely, red (660 nm), green (550 nm), red-edge (735 nm), and near-infra-red (790 nm), and an RGB sensor. The application “Tower” was used to automate the flight mission with a front and side overlap of 75% each, at a speed of 5 m/s, and conducted at 40 m above ground level to estimate NDVI. These flight parameters delivered high-resolution orthomosaics with a ground sampling distance of 2.69 cm. The acquired images were processed using the software Pix4D mapper Pro (Pix4D, Lausanne, Switzerland) to generate high-resolution orthomosaics and NDVI maps (Gebremedhin et al., 2020). In brief, the images were imported into the Pix4D software and radiometrically corrected using a known reflectance source, the Airinov calibration plate (Airinov, Paris, France), to generate orthomosaics and NDVI TIFF images. These TIFF files were imported into ArcGIS Pro version 2.1 (https://www.arcgis.com/) to estimate plot level NDVI values. The rectangular polygons with a unique number (plot number) were created and overlaid on the NDVI orthomosaics image to extract plot level average NDVI values for each trial.

### Genotyping

Eight seeds from each line of field pea were germinated, leaf tissue samples of five seedlings were pooled, and total RNA was extracted using RNeasy^®^ 96 Kit (Qiagen, Hilden, Germany). RNA-Seq libraries were prepared using SureSelect stranded RNA library preparation kit (Agilent Technologies, Santa Clara, CA) following the manufacturer's instructions (Malmberg et al., 2018), and sequencing was performed on either the HiSeq 3000 or Novaseq system (Illumina Inc., San Diego, USA) to a depth of 4–5 million paired reads per sample. The sequence reads were filtered and aligned to the field pea reference genome, Pisum_sativum_v1a.fa of cultivar Cameor (Kreplak et al., 2019). Variant calling was performed using GATK (Van der Auwera et al., 2013), and SNP filtering was performed with read depth (DP ≥ 5), missing rate (<60%), and base quality (Q30). Genotype imputation was performed using the Beagle package (Browning and Browning, 2007). A total of 1,453 lines with 47,352 SNPs (40% heterozygosity) were used for genomic prediction.

The genomic relationship matrix (GRM) was calculated for all lines according to VanRaden (2008). Each breeding population GRM was extracted from the combined GRM. A heatmap sorted by hierarchical clustering based on the GRM in each breeding cycle was combined and plotted in R. The principal component analysis (PCA) based on combined GRM was performed to visualize cluster information across the breeding cycles.

### Statistical Analysis

All statistical analyses were conducted in ASReml (Gilmour et al., 2015), and variance components were estimated with implemented restricted maximum likelihood (REML) method.

#### Univariate Models

The general form of the univariate linear mixed model was as follows:


(1) $$y\;=\;Xb\;+\;Z_{\text{g}}g\;+\;Z_{\text{r}}r\;+\;Z_{\text{c}}c\;+\varepsilon$$

where **y** is a vector of phenotypic values for each line, and **b** is a vector of fixed effects, including the mean and replications. For the basic best linear unbiased prediction (BLUP) model, **g** is a vector of random genetic effects of lines ~ *N* (0, **I**$I\sigma_{G}^{2}$), **I** is an identity matrix, and $\sigma_{G}^{2}$ is the genetic variance due to lines; for the additive genetic model with pedigree information, **g** is a vector of random additive genetic effects explained by pedigree, and **I** is replaced with **A**, a numerator relationship matrix based on pedigree, referred to as ABLUP; with **G**, a genomic relationship matrix calculated from SNP markers, referred as GBLUP; with fitting **A** and **G** simultaneously, referred as AGBLUP. $\sigma_{a}^{2}$ is the additive genetic variance captured by pedigree; $\sigma_{g}^{2}$ is the additive genetic variance explained by markers; **r** and **c** are the vectors of random field design effects for row and column; **ε** includes the independent measurement residual ~ *N* (0, **I**$\sigma_{e}^{2}$), and the spatial dependent residual, which includes the row and column two-dimensional covariance, $R=\sum_{r}^{}\left(p_{r}\right)⊗\sum_{c}^{\;}\left(p_{c}\right).$. **X**, **Z**_**g**_, **Z**_**r**_, and **Z**_**c**_ are the incidence matrices associating phenotypes with fixed and random effects of **b**, **g**, **r**, and **c**, respectively. The non-additive genetic component was also estimated by fitting line effects in addition to pedigree and marker effects in model 1. We assumed that the variance explained by pedigree and marker effects were associated with additive effects, and the remaining genetic variance explained by lines was due to non-additive genetic effects.

Best linear unbiased estimates (BLUE) were calculated using a univariate model similar to the BLUP model, but the breeding lines were fitted as fixed effects. BLUEs were used as adjusted phenotypic data to calculate the genomic PA in different models (Supplementary Table 2).

#### Multivariate Models

The multivariate model with three traits, also referred to as the BLUP multivariate model, was illustrated as follows:


(2) $$\begin{array}{l} \left[\begin{array}{c} y_{1}\\ y_{2}\\ y_{3} \end{array}\right]=\left[\begin{array}{ccc} X_{1} & 0 & 0\\ 0 & X_{2} & 0\\ 0 & 0 & X_{3} \end{array}\right]\left[\begin{array}{c} b_{1}\\ b_{2}\\ b_{3} \end{array}\right]+\left[\begin{array}{ccc} Z_{g1} & 0 & 0\\ 0 & Z_{g2} & 0\\ 0 & 0 & Z_{g3} \end{array}\right]\;\left[\begin{array}{c} g_{1}\\ g_{2}\\ g_{3} \end{array}\right]+\\ \;\left[\begin{array}{ccc} Z_{r1} & 0 & 0\\ 0 & Z_{r2} & 0\\ 0 & 0 & Z_{r3} \end{array}\right]\left[\begin{array}{c} r_{1}\\ r_{2}\\ r_{3} \end{array}\right]+\left[\begin{array}{ccc} Z_{c1} & 0 & 0\\ 0 & Z_{c2} & 0\\ 0 & 0 & Z_{c3} \end{array}\right]\left[\begin{array}{c} c_{1}\\ c_{2}\\ c_{3} \end{array}\right]+\;\left[\begin{array}{c} \varepsilon_{1}\\ \varepsilon_{2}\\ \varepsilon_{3} \end{array}\right] \end{array}$$

where the model terms for traits 1, 2, and 3 were similar to the previously described univariate model. In this model, $\left[\begin{array}{c} g_{1}\\ g_{2}\\ g_{3} \end{array}\right]\;\sim \;N\left(0,\;I⊗T\right)$ where $T=\;\left[\begin{array}{ccc} \sigma_{g1}^{2} & \sigma_{g12} & \sigma_{g13}\\ \sigma_{g12} & \sigma_{g2}^{2} & \sigma_{g23}\\ \sigma_{g13} & \sigma_{g23} & \sigma_{g3}^{2} \end{array}\right]$ is the variance-covariance matrix of three traits; and

$\left[\begin{array}{c} \varepsilon_{1}\\ \varepsilon_{2}\\ \varepsilon_{3} \end{array}\right]\;\sim \;N\left(0,\;I⊗R\right)$ where $R=\;\left[\begin{array}{ccc} \sigma_{e1}^{2} & \sigma_{e12} & \sigma_{e13}\\ \sigma_{e12} & \sigma_{e1}^{2} & \sigma_{e23}\\ \sigma_{e13} & \sigma_{e23} & \sigma_{e1}^{2} \end{array}\right]$ is the independent residual variance and covariance matrix for three traits as described for the univariate model. **I** is the incidence matrix. When **I** was replaced with **G**, the model was referred to GBLUP multivariate model.

#### Heritability and Genetic Correlation

Broad-sense heritability (*H*^2^) for each trait was calculated as the proportion of phenotypic variance explained by genetic variance ($\sigma_{G}^{2}$), which was estimated with univariate models; where $\sigma_{G}^{2}$ is the genetic variance of lines, $\sigma_{e}^{2}$ is the random independent residual error variance, and rep is the trial replicates number (Holland et al., 2002).


$$\begin{array}{l} {\text{H}}^{2}=\sigma_{G}^{2}/\left({\;\sigma }_{G}^{2}+\sigma_{e}^{2}/rep\right) \end{array}$$

Narrow-sense heritability (*h*^2^) was represented as $h_{a}^{2}$, narrow-sense heritability captured by pedigree; $h_{g}^{2}$, narrow-sense heritability captured by markers; $h_{a+g}^{2}$ , narrow-sense heritability captured by pedigree and markers, and they were estimated as follows:


$$\begin{array}{l} h_{a}^{2}=\sigma_{a}^{2}/\left(\sigma_{a}^{2}+\sigma_{e}^{2}/rep\right), \end{array}$$

$$\begin{array}{l} h_{g}^{2}=\sigma_{g}^{2}/\left(\sigma_{g}^{2}+\sigma_{e}^{2}/rep\right), \end{array}$$

$$\begin{array}{l} h_{\left(a\;+\;g\right)}^{2}\;=\;\left(\sigma_{a}^{2}\;+\;\sigma_{g}^{2}\right)/\left(\sigma_{a}^{2}+\sigma_{g}^{2}\;+\;\sigma_{e}^{2}/rep\right), \end{array}$$

where $\sigma_{a}^{2}$ is the additive genetic variance explained by pedigree (**A**), and $\sigma_{g}^{2}$ is the additive genetic variance explained by makers (**G**). When non-additive genetic effects were decomposed, narrow-sense heritability could be estimated the same way but the variance of line effects was added to the total phenotypic variance.

Genetic correlation (*r*_*G*_) between traits was calculated using the variance components estimated from BLUP multivariate models in four breeding populations, where $\sigma_{Gi}^{2}$ and $\sigma_{Gj}^{2}$ are the total genetic variance of traits i and j, and $\sigma_{Gij}^{2}$ is the total genetic covariance between traits i and j.


$$\begin{array}{l} r_{G}=\frac{\sigma_{Gi,j}}{\sqrt{\sigma_{Gi}^{2}\sigma_{Gj}^{2}}}\; \end{array}$$

In GBLUP multivariate model, the additive genetic correlation (*r*_*g*_) between traits was calculated in the same way but with additive genetic variance, and covariance of traits was based on **G**.

### Genomic Prediction and Validation

The genomic prediction was evaluated for grain yield with the GBLUP univariate model and GBLUP multivariate models, including fitting both the NDVI and yield together as a bivariate model and fitting NDVI, BBSC, and yield as a multivariate model. The PA was defined as Pearson's correlation coefficient between genomic estimated breeding values (GEBVs) and BLUEs. In the cross-validation method, all lines within each breeding cycle were randomly divided into five equal subsets. Each subset was, in turn, chosen as the validation set and was subsequently predicted by using the other four subsets as the training set. The cross-validation process was repeated five times, and mean PA and standard deviation (SD) were calculated across all 25 validation sets. In the forward prediction method, the training set consisted of one or multiple breeding stages previous to the validation breeding stage. A total of six scenarios were tested using GBLUP models, including using traits in the S0 to independently predict S1, S2, and S3; combining S0 and S1 to predict S2 and S3; and combining S0, S1, and S2 to predict S3.

## Results

### Summary Statistics of the Traits

**Figure 1:**
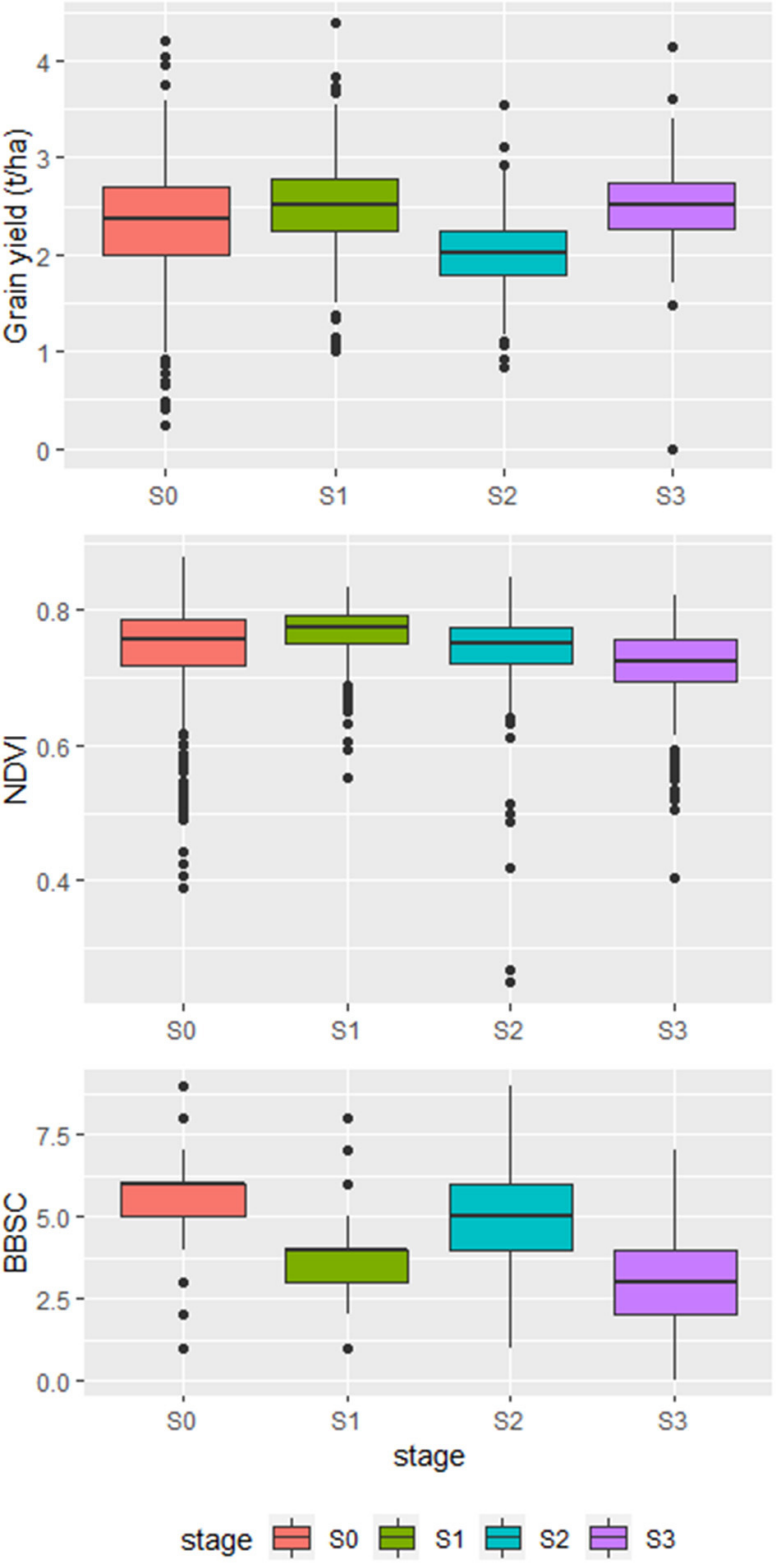
Box plots of grain yield, NDVI, and BBSC across the breeding stages (S0–S3).

Summary statistics of grain yield, NDVI, and BBSC for the four breeding stages are shown in Figure 1. The average grain yield varied across the stages. S2 had the lowest average grain yield, while the other three stages had average grain yields of ≈2.5 t/ha. The average NDVI was consistent, and S3 showed the lowest NDVI. The average BBSC varied dramatically across stages. S1 and S3 showed lower BBSC than S0 and S2.

### Population Structure

**Figure 2:**
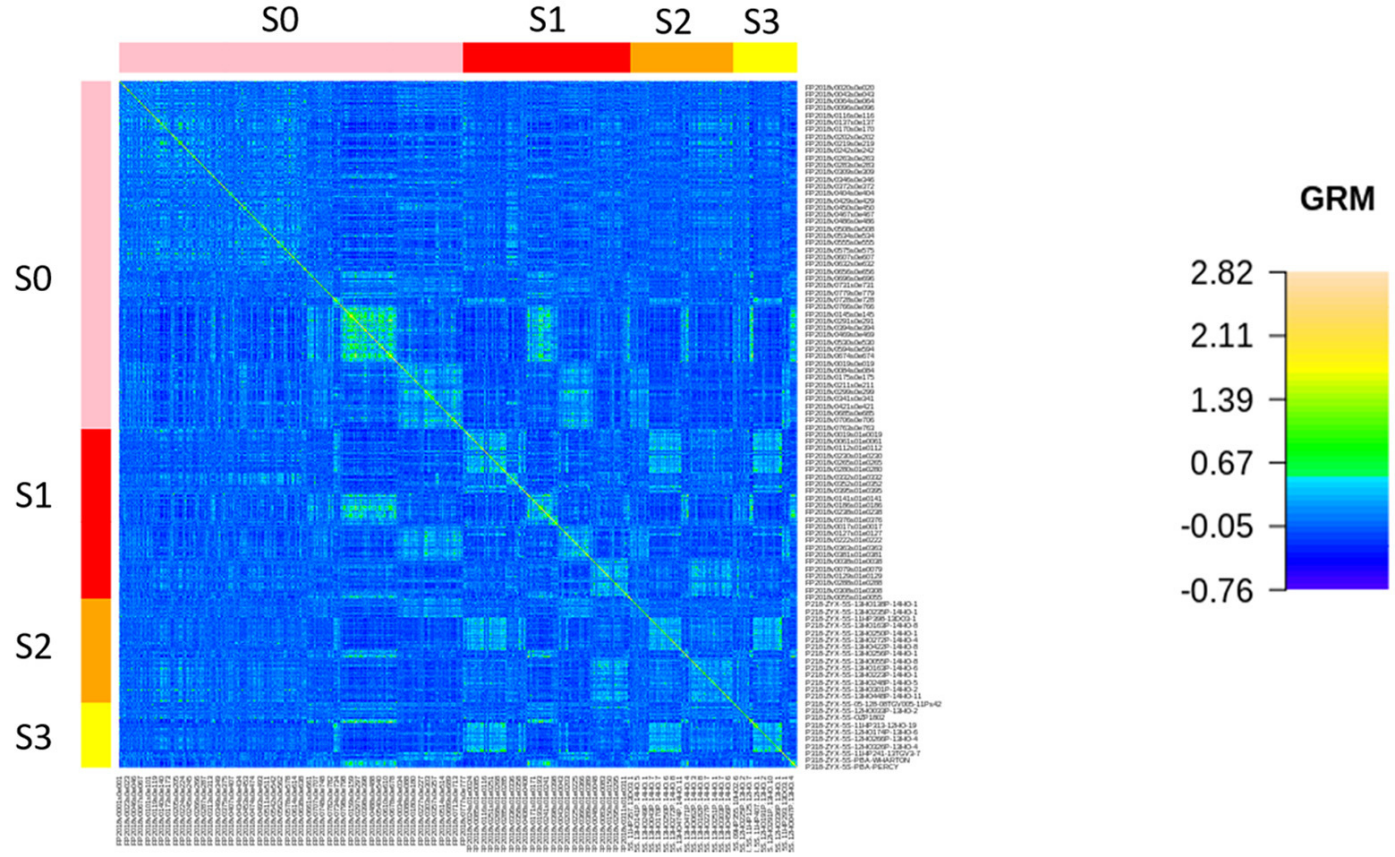
Heatmap of the genomic relationship matrix (GRM) for field pea breeding stages represented with differed colors (S0_pink, S1_red, S2_orange, and S3_yellow). The colors within the GRM indicate the degree of relatedness between breeding lines (high relationships are shown in green, and low relationships are shown in blue).

In total, 1,453 breeding lines with 47,352 SNPs were used to calculate the GRM. With all stages combined, three groups could be observed according to the dendrogram with different levels of relatedness among lines within and between the groups (Supplementary Figure 2). When the GRM was ordered according to breeding stages, highly related lines across stages were observed (Figure 2). S0 was the largest breeding stage and had 738 lines with genotypic data. Two main subgroups of closely related individuals within S0 had close relatives in S1, S2, and S3 (Figure 2, Supplementary Figure 3). There were 356 genotyped lines in S1, and some of them had close relationships with each other. One of the highly related S1 subgroups had close relatives in S2 and S3 but not in S0 (Figure 2, Supplementary Figure 3). In S2 and S3, 223 and 136 lines were genotyped, respectively. The heatmaps for the subset of the GRM for each stage clearly showed more highly related lines within S3 than S1 (Supplementary Figure 3). According to PCA based on the GRM, the first three PCs explained nearly 70% of the total variance, and the plots for 1st, 2nd, and 3rd PCs further showed that all stages of the breeding lines generally overlapped (Supplementary Figure 4).

### Variance Components and Heritability

Estimating variance components from different breeding stages will help us understand the breeding population, especially when selection is conducted at each breeding cycle. The genetic parameters estimated for each trait would help us to observe the traits' genetic architecture. Overall, the non-additive genetic variance was observed for all three traits, but its proportion varied with models fitted and varied across breeding stages. The estimated *h*^2^ was reduced in most cases for all traits when including non-additive genetic components while the estimated error variances were not reduced. The proportion of total genetic variation explained by additive genetic effects, represented as the estimated $h_{g}^{2}$ (by marker), was comparable to $h_{a}^{2}$ (by pedigree) when the non-additive genetic variance was not included.

When combining all breeding stages together, the estimated broad-sense heritability (*H*^2^) was 0.58, 0.47, and 0.38 for grain yield, NDVI, and BBSC, respectively (Table 1). The estimated narrow-sense heritability (*h*^2^, represented as $h_{a}^{2}$, $h_{g}^{2}$, and $h_{\left(a+g\right)}^{2}$) was lower than *H*^2^ for combined stages and each breeding stage except for NDVI. The estimated *h*^2^ for grain yield ranged from 0.33 to 0.52, and the estimates were reduced and ranged from 0.25 to 0.46 when line effects were included in the model for stages 0–2. Stage 3 showed higher estimated *h*^2^ when line effects were not included. The estimated *h*^2^ value for NDVI was moderate, ranging from 0.33 to 0.50 without including line effects. Including line effects in the model reduced the *h*^2^ dramatically in S1. S3 showed the highest estimated *h*^2^ compared with other stages. The estimated *h*^2^ for BBSC was not reduced much when including and excluding line effects for S0 to S2, ranging from 0.26 to 0.4; however, the *h*^2^ estimated for S3 was lower than that in other stages.

**Table 1:** Total genetic variance ($\sigma_{G}^{2}$), additive genetic variance ($\sigma_{a}^{2}$ or $\sigma_{g}^{2}$), and non-additive genetic variance ($\sigma_{Gl}^{2}$) of grain yield, NDVI, and BBSC at each breeding stage, and broad (H^2^) and narrow-sense heritability (h^2^) and their standard error (SE) from model 1—univariate models.

		**Grain yield**					**NDVI**					**BBSC**				
		**S0–3**	**S0**	**S1**	**S2**	**S3**	**S0–3**	**S0**	**S1**	**S2**	**S3**	**S0–3**	**S0**	**S1**	**S2**	**S3**
Line	σG	0.088	0.11	0.06	0.045	0.06	0.0009	0.0014	0.0003	0.0004	0.001	0.44	0.54	0.28	0.41	0.32
	σe	0.13	0.16	0.1	0.07	0.05	0.002	0.002	0.0008	0.0008	0.002	1.46	0.71	0.88	0.80	1.4
	${\text{H}}_{\text{G}}^{2}$	0.58	0.51	0.55	0.56	0.71	0.47	0.51	0.43	0.5	0.5	0.38	0.53	0.39	0.51	0.31
	SE	0.02	0.04	0.04	0.05	0.06	0.02	0.04	0.03	0.05	0.06	0.02	0.05	0.03	0.05	0.05
Pedigree	σa	0.061	0.07	0.04	0.03	0.04	0.0006	0.0007	0.0003	0.0003	0.0007	0.28	0.29	0.19	0.31	0.20
	σe	0.13	0.16	0.09	0.07	0.05	0.002	0.002	0.0008	0.0008	0.002	1.35	0.68	0.84	0.81	1.46
	h_*a*_^2^	0.48	0.4	0.47	0.46	0.62	0.38	0.34	0.43	0.43	0.41	0.29	0.39	0.31	0.43	0.22
	SE	0.02	0.04	0.03	0.05	0.06	0.02	0.04	0.03	0.04	0.05	0.02	0.04	0.03	0.04	0.04
Pedigree + line	σa	0.04	0.06	0.04	0.03	0.022	0.0005	0.0006	0.0001	0.0003	0.0007	0.26	0.29	0.19	0.31	0.13
	σGl	0.02	0.016	0	0	0.023	0.0002	0.0002	0.0002	0	0	0.03	0	0	0	0.14
	σe	0.13	0.16	0.09	0.07	0.05	0.002	0.002	0.0008	0.0008	0.002	1.35	0.68	0.84	0.81	1.42
	h_*a*_^2^	0.32	0.33	0.47	0.46	0.31	0.29	0.28	0.14	0.43	0.41	0.27	0.39	0.31	0.43	0.13
	SE	0.04	0.05	0.03	0.05	0.12	0.03	0.05	0.05	0.04	0.12	0.02	0.04	0.03	0.04	0.04
Marker	σg	0.076	0.06	0.04	0.03	0.06	0.0009	0.0009	0.0002	0.0004	0.001	0.39	0.32	0.16	0.31	0.31
	σe	0.14	0.18	0.1	0.08	0.05	0.002	0.002	0.0008	0.0008	0.002	1.37	0.76	0.85	0.81	1.42
	h_*g*_^2^	0.52	0.33	0.44	0.43	0.71	0.47	0.4	0.33	0.5	0.5	0.36	0.39	0.27	0.43	0.3
	SE	0.03	0.04	0.04	0.06	0.07	0.03	0.04	0.04	0.05	0.06	0.02	0.04	0.03	0.05	0.05
Marker + line	σg	0.047	0.05	0.03	0.02	0.02	0.0006	0.0008	0.0001	0.0004	0.0008	0.27	0.3	0.16	0.25	0.2
	σGl	0.031	0.04	0.009	0.01	0.04	0.0003	0.0004	0.0002	0	0.0001	0.1	0.05	0.01	0.09	0.12
	σe	0.12	0.16	0.1	0.07	0.05	0.0015	0.002	0.0008	0.0008	0.002	1.32	0.73	0.84	0.79	1.4
	h_*g*_^2^	0.34	0.25	0.34	0.31	0.24	0.36	0.32	0.14	0.5	0.42	0.26	0.36	0.27	0.34	0.2
	SE	0.03	0.04	0.05	0.07	0.08	0.03	0.04	0.04	0.05	0.1	0.02	0.04	0.04	0.05	0.07
Pedigree + marker	σa	0.027	0.028	0.014	0.018	0.036	0.0003	0.0003	0.0001	0.0001	0.0002	0.09	0.1	0.005	0.1	0.08
	σg	0.04	0.043	0.025	0.016	0.004	0.0005	0.0006	0.0001	0.0002	0.0006	0.24	0.23	0.16	0.22	0.2
	σe	0.13	0.16	0.1	0.07	0.05	0.002	0.002	0.0008	0.0008	0.002	1.3	0.69	0.85	0.8	1.41
	h_*a*+*g*_^2^	0.51	0.4	0.44	0.49	0.62	0.44	0.4	0.33	0.43	0.44	0.34	0.42	0.28	0.44	0.28
	SE	0.03	0.04	0.04	0.05	0.06	0.03	0.04	0.04	0.05	0.06	0.02	0.04	0.03	0.05	0.05
Pedigree + marker + line	σa	0.013	0.02	0.014	0.016	0.02	0.0003	0.0003	0.00004	0.0001	0.0002	0.076	0.1	0.002	0.083	0.062
	σg	0.038	0.04	0.025	0.016	0.003	0.0005	0.0001	0.0001	0.0002	0.0006	0.24	0.23	0.15	0.21	0.18
	σGl	0.02	0.02	0	0.002	0.02	0.00006	0.0001	0.0001	0	0	0.025	0	0.008	0.015	0.05
	σe	0.12	0.16	0.1	0.07	0.05	0.0015	0.002	0.0008	0.0008	0.002	1.3	0.69	0.84	0.79	1.4
	h_*a*+*g*_^2^	0.39	0.32	0.44	0.46	0.34	0.5	0.22	0.22	0.43	0.44	0.32	0.42	0.26	0.42	0.24
	SE	0.03	0.05	0.04	0.1	0.11	0.04	0.05	0.05	0.05	0.06	0.02	0.04	0.04	0.07	0.08

### Genetic Correlation

The BLUP and GBLUP multivariate models showed nearly identical correlation patterns between traits (Table 2). In S0, S1, and S2, grain yield showed a strong positive correlation with NDVI both in the BLUP (0.75–0.89) and GBLUP (0.78–0.85), and a strong negative correlation with BBSC in the BLUP (−0.72 to −0.76) and in the GBLUP (−0.70 to −0.78). The correlation between NDVI and BBSC was also strong, ranging from −0.84 to −0.94 in the BLUP model and −0.76 to −0.88 in the GBLUP model. In S3, the correlation among traits was lower except for the strong correlation between yield and NDVI in the BLUP model (0.83). The standard errors associated with the estimated correlations were much higher in S3 compared with other stages.

**Table 2:** The genetic correlation and the standard error (SE) between traits estimated with BLUP and GBLUP models within each breeding stage.

		**Yield to NDVI**		**Yield to BBSC**		**NDVI to BBSC**	
	**Stage**	**rG**	**SE**	**rG**	**SE**	**rG**	**SE**
BLUP	S0	0.80	0.057	−0.76	0.07	−0.84	0.07
	S1	0.75	0.10	−0.72	0.135	−0.94	0.15
	S2	0.89	0.10	−0.75	0.096	−0.86	0.11
	S3	0.83	0.17	−0.35	0.18	−0.13	0.25
GBLUP	S0	0.78	0.058	−0.78	0.065	−0.88	0.042
	S1	0.79	0.06	−0.70	0.078	−0.76	0.072
	S2	0.85	0.07	−0.78	0.076	−0.86	0.07
	S3	0.14	0.19	−0.44	0.23	−0.19	0.27

### Genomic Prediction Accuracy

Apart from the S3, the PA for grain yield with the cross-validation method was higher in the bivariate model, which combined yield and NDVI compared with univariate models (Table 3). The difference in PA was more dramatic between univariate and bivariate models compared with bivariate and multivariate models, which combined yield, NDVI, and BBSC. Using the bivariate model, in S0, the PA improved from 0.41 to 0.60, a nearly 50% increase, while in S2, the accuracy improved from 0.39 to 0.49, about a 25% increase. In S1, the PA increased from 0.5 to 0.53, about a 6% increase with the bivariate model. The PA was low in S3, and the highest PA of 0.28 was achieved with the univariate model.

**Table 3:** The mean and standard deviation (sd) of PA for grain yield within each breeding stage in the cross-validation method with the univariate, bivariate, and multivariate models.

		**Univariate model**		**Bivariate model**		**Multivariate model**	
	**Lines**	**Mean**	**SD**	**Mean**	**SD**	**Mean**	**SD**
S0	738	0.41	0.07	0.60	0.05	0.61	0.04
S1	356	0.50	0.08	0.53	0.07	0.53	0.09
S2	223	0.39	0.14	0.49	0.13	0.54	0.13
S3	136	0.28	0.11	0.26	0.15	0.23	0.15

**Figure 3:**
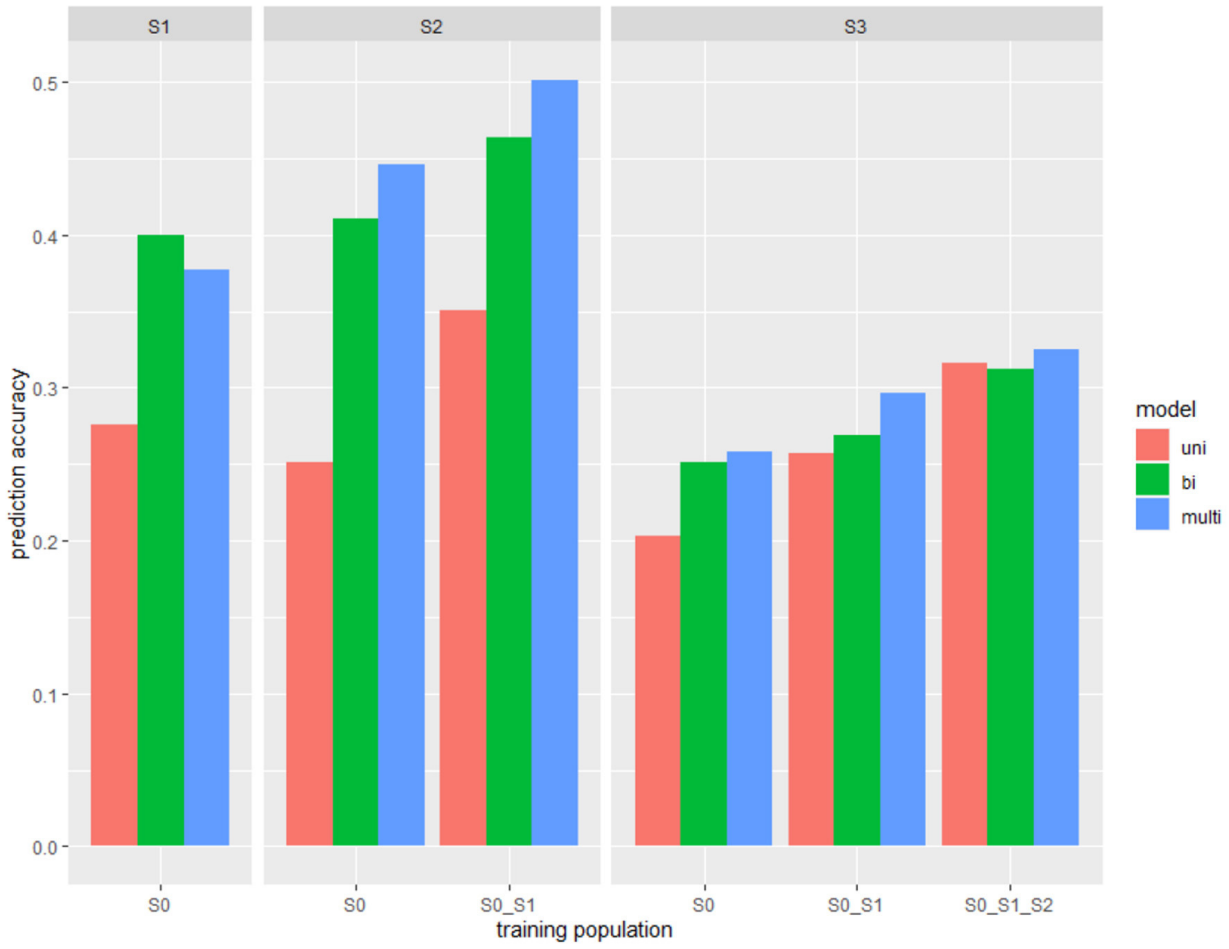
The PA for forward prediction scenarios where one or multiple breeding stages were used as the training set to predict the grain yield in the next breeding stage with different models (uni, univariate; bi, bivariate; multi, multivariate).

The PA was generally lower in the forward validation method compared with cross-validation except for S3 when S0–S2 were used as a training set (Figure 3). Using S0 as a training population to predict the yield in S1, S2, or S3, the bivariate model dramatically increased the PA, about 60% improvement was observed to predict S2. When combining breeding populations, using S0 and S1 to predict S2 or S3, PA was increased in bivariate and multivariate models. When S0, S1, and S2 were combined and used to predict S3, all models showed PA above 0.3, whereas the multivariate model had the highest PA (0.33).

## Discussion

Increasing the grain yield in field pea could increase the crop's economic return and attract farmers to grow the crop. GS is a modern breeding approach that could substantially improve the rate of genetic gain for grain yield. Its deployment depends on the PA that can be achieved. Traits that are correlated with grain yield have the potential to improve PA through the incorporation into the GS models. In this study, we investigate combining NDVI and BBSC in multivariate models to improve PA of grain yield in field pea.

The univariate models have been applied in pea GS studies, and PA for seed yield within the population reached 0.3 with the Bayesian Lasso model and 0.42 with the Reproducing kernel Hilbert space model (Annicchiarico et al., 2019; Bari et al., 2021). Using the GBLUP multivariate model, we achieved higher PA, around 0.5 within the population and above 0.4 when using the forward prediction method for grain yield, supporting that the multivariate model could improve PA in pea genome prediction. We observed close to a 50% PA increase when using bivariate models to predict grain yield, while a study in wheat reported about 70% increase in PA with the multivariate models (Rutkoski et al., 2016). In our study, NDVI and BBSC are both highly correlated with yield in S0–S2 stages. However, the correlation among traits in S3 was low. The reason was not very clear, but the small population size, close relationship between lines, and high inbreeding could impact the correlation pattern. Within S3, the PA of yield was lower in the multivariate model than the univariate model, suggesting that correlations between traits were important in multivariate models to improve PA. Similar conclusions were also observed with the multivariate studies in barley and wheat (Hayes et al., 2017; Bhatta et al., 2020). However, a sorghum GS study showed that high heritability with low correlations could also improve the PA when combined with multivariate models (Velazco et al., 2019). We observed that when we added the third highly correlated trait, BBSC, into the model, PA improvement was limited compared with the bivariate model. This is likely due to the high correlation between NDVI and BBSC and the low heritability of BBSC, resulting in less additional information to further improve the predictions.

The training population size is critical in achieving sound PA, and this can be an issue in smaller breeding programs that are not well resourced. Including the previous generation in the training population is an effective way to increase the PA, and this has been shown in several studies (Auinger et al., 2016; Gill et al., 2021). In the current pea breeding program, the breeding materials were descendants of more than 100 founder lines (Supplementary Table 1). The recycling of highly adapted parents in the crossing program induced the inbred nature of the current breeding population, although Burstin et al. (2015) highlighted the existence of large genetic diversity within the *Pisum sativum* genepool. All the breeding lines in different breeding stages have gone through different selection cycles. For example, breeding lines in advanced trials such as S2 and S3 were selected based on yield data and phenology, disease resistance, and abiotic stress tolerance from multi-location environment trials. In contrast, breeding lines in early-stage trials such as S0 and S1 were selected based on limited data and came through fewer cycles of selection. When combining early breeding stages to increase the reference population size, the relatedness between lines and diverse selection environments could be beneficial for predicting the later breeding cycle. We observed a higher PA in all the forward prediction scenarios, especially in S3, and was higher than the PA that cross-validation achieved when all the previous stages were combined and used as the training population. This supports the work of Bari et al. (2021), which showed that an increase in the size of the reference population could increase PA and reported similar PA (0.27–0.48) for pea yield.

The heritability of traits is important in GS and could affect the PA as genomic prediction depends on the precisely estimated SNP effects and the proportion of the variance explained by the SNPs (Daetwyler et al., 2008; Goddard, 2009). Narrow sense heritability (*h*^2^) can be estimated using the pedigree information, the proportion of variance explained by numerator **A** matrix (Oakey et al., 2006), or using the molecular marker information, the proportion of variance explained by **G** matrix (Goddard et al., 2011). Our study estimated variance components and *h*^2^ with ABLUP, GBLUP, and AGBLUP models. The additive genetic variance explained by markers was comparable with the pedigree, indicating that SNPs used in our study could reflect the similarity between lines. However, a proportion of the genetic variance could not be captured by **G** when comparing the GBLUP model with the AGBLUP model. This could be due to some causal mutations segregated within families or lines, which were not captured by the markers (Khansefid et al., 2014). In our study, we also fitted the non-additive genetic effects in the model, and we observed a reduction in the estimated *h*^2^. In contrast, the estimated error variance was not reduced with or without the line effects. This indicated that a proportion of variance explained by line could be absorbed as additive genetic variance in the ABLUP and GBLUP models, thereby inflating the estimation of *h*^2^. Early studies supported those observations (Kumar et al., 2015; Piaskowski et al., 2018). The impact of the non-additive genetic effects on PA needs to be further studied.

Pea grain yield has been associated with rainfalls (annual and growing season rainfall), minimum temperatures early in the season, and maximum temperatures during seed filling. In Australia, ≈70% of the field pea crop is grown in low rainfall (<230 mm) environments (Leonforte et al., 2013). In 2018, the season was relatively dry, receiving only 157 mm of rainfall during the entire growing season. Maximum and minimum temperatures were cooler early in the season, and a substantial number of frost hours (hours below freezing point) were recorded throughout the season (Supplementary Figure 1), encouraging the early season bacterial blight outbreak. Further, the lack of follow-up rains meant that crops did not recover, and substantial bacterial blight losses were observed in the farmer's field. High correlations between yield and BBSC within the breeding population reflected the impact of bacteria blight on grain yield in 2018. This severe bacterial blight epidemic was not seen in other breeding program trials. Although it is a limitation of this study with only 1 year of data in one location, the use of four trials with different population sizes allowed the investigation of traits' correlation and the development of useful prediction equations. Currently, phenotyping of yield and disease traits in the breeding program can be a challenging task due to the time and resource demands and the subjective nature of measurements. As an important auxiliary trait in predicting grain yield, NDVI has been used to predict yield in wheat (Sun et al., 2017; Hassan et al., 2019). Our study revealed a high correlation between NDVI and grain yield, consistent with the recent study in pea observing a similarly high correlation, 0.85, without biotic stresses (Zhang et al., 2021). High correlations between NDVI and BBSC further support the effectiveness of using NDVI in predicting pea grain yield. Our study indicated that at an early stage, the prediction of grain yield with HTP in field pea is feasible.

In conclusion, this study estimated the heritability for grain yield, NDVI, and BBSC in a field pea breeding program, and it also evaluated the genomic PA for grain yield with univariate and multivariate models. The results showed that heritability for all traits was low to moderate. However, strong genetic correlations were observed among traits apart from S3. In both cross-validation and forward prediction methods, we found that the multivariate model outperformed the univariate models by substantially improving PA. We also confirmed that with a larger training set, a higher PA could be achieved when different breeding stages were included in the training population. Field phenotyping is costly and labor-intensive for large breeding trials. Both NDVI and BBSC are commonly used traits in breeding programs that could be measured in the early growth stage. Our study has shown the potential of adopting NDVI with multivariate models to improve grain yield PA. GS is becoming popular among crop breeders to predict phenotypes, especially yield. We showed that GS holds great potential for improving grain yield in field pea breeding.

## Data Availability Statement

The details of the data and results supporting the discussions and conclusions are included in the Supplementary Materials. The genotype data used for this study are deposited in the figshare repository, can be accessed from here: https://doi.org/10.6084/m9.figshare.19817509.

## Author Contributions

HZ, BP, SKant, and GR conceived and designed the experiment. BP, SS, SJ, SKant, and Skaur performed and supervised the phenotyping and genotyping. HZ performed the statistical analysis and wrote the draft. BP and MK assisted in the data analysis and interpretation. SKaur and GR supported the study. All authors revised the manuscript.

## Funding

This study was funded by Agriculture Victoria Research and Grains Research Development Corporation through a research project DAV00153.

## Conflict of Interest

The authors declare that the research was conducted in the absence of any commercial or financial relationships that could be construed as a potential conflict of interest.

## Publisher's Note

All claims expressed in this article are solely those of the authors and do not necessarily represent those of their affiliated organizations, or those of the publisher, the editors and the reviewers. Any product that may be evaluated in this article, or claim that may be made by its manufacturer, is not guaranteed or endorsed by the publisher.

## References

[B1] Amarakoon D. Thavarajah D. McPhee K. Thavarajah P. (2012). Iron-, zinc-, and magnesium-rich field peas (*Pisum sativum* L.) with naturally low phytic acid: a potential food-based solution to global micronutrient malnutrition. J. Food Compos. Anal. 27, 8–13. 10.1016/j.jfca.2012.05.007

[B2] Annicchiarico P. Nazzicari N. Pecetti L. Romani M. Russi L. (2019). Pea genomic selection for Italian environments. BMC Genom. 20:603. 10.1186/s12864-019-5920-x 31331290 PMC6647272

[B3] Auinger H. J. Schonleben M. Lehermeier C. Schmidt M. Korzun V. Geiger H. H. (2016). Model training across multiple breeding cycles significantly improves genomic prediction accuracy in rye (*Secale cereale* L.). Theor. Appl. Genet. 129, 2043–2053. 10.1007/s00122-016-2756-5 27480157 PMC5069347

[B4] Bao Y. Kurle J. E. Anderson G. Young N. D. (2015). Association mapping and genomic prediction for resistance to sudden death syndrome in early maturing soybean germplasm. Mol. Breed 35, 128. 10.1007/s11032-015-0324-3 25999779 PMC4434860

[B5] Bari M. A. A. Zheng P. Viera I. Worral H. Szwiec S. Ma Y. (2021). Harnessing genetic diversity in the USDA pea germplasm collection through genomic prediction. Front. Genet. 12:707754. 10.3389/fgene.2021.707754 35003202 PMC8740293

[B6] Bashi Z. McCullough R. Ong L. Ramirez M. (2019). Alternative Proteins: The Race for Market Share is on. Protein report. Available online at: https://www.mckinsey.com/industries/agriculture/our-insights/alternative-proteins-the-race-for-market-share-is-on

[B7] Bhatta M. Gutierrez L. Cammarota L. Cardozo F. German S. Gomez-Guerrero B. (2020). Multi-trait genomic prediction model increased the predictive ability for agronomic and malting quality traits in barley (*Hordeum vulgare* L.). G3 10, 1113–1124. 10.1534/g3.119.400968 31974097 PMC7056970

[B8] Bretag T. W. Keane P. J. Price T. V. (2006). The epidemiology and control of ascochyta blight in field peas: a review. Aust. J. Agric. Res. 57, 883–902. 10.1071/AR05222

[B9] Browning S. R. Browning B. L. (2007). Rapid and accurate haplotype phasing and missing-data inference for whole-genome association studies by use of localized haplotype clustering. Am. J. Hum. Genet. 81, 1084–97. 10.1086/521987 17924348 PMC2265661

[B10] Burstin J. Gallardo K. Mir R. Varshney R. Duc G. Burstin J. (2011). “Improving protein content and nutrition quality,” in Biology and Breeding of Food Legumes, eds A. Pradeep and J. Kumars. 10.1079/9781845937669.0314

[B11] Burstin J. Salloignon P. Chabert-Martinello M. Magnin-Robert J. B. Siol M. Jacquin F. (2015). Genetic diversity and trait genomic prediction in a pea diversity panel. BMC Genom. 16:105. 10.1186/s12864-015-1266-1 25765216 PMC4355348

[B12] Cheng P. Holdsworth W. Ma Y. Coyne C. J. Mazourek M. Grusak M. A. (2015). Association mapping of agronomic and quality traits in USDA pea single-plant collection. Mol. Breed. 35, 75. 10.1007/s11032-015-0277-631850030

[B13] Coyne C. J. Kumar S. Wettberg E. J. B. Marques E. Berger J. D. Redden R. J. (2020). Potential and limits of exploitation of crop wild relatives for pea, lentil, and chickpea improvement. Legume Sci. 2, e36. 10.1002/leg3.36

[B14] Crossa J. Perez-Rodriguez P. Cuevas J. Montesinos-Lopez O. Jarquin D. de Los Campos G. (2017). Genomic selection in plant breeding: methods, models, and perspectives. Trends Plant Sci. 22, 961–975. 10.1016/j.tplants.2017.08.011 28965742

[B15] Daetwyler H. D. Villanueva B. Woolliams J. A. (2008). Accuracy of predicting the genetic risk of disease using a genome-wide approach. PLoS ONE 3:e3395. 10.1371/journal.pone.0003395 18852893 PMC2561058

[B16] de Los Campos G. Hickey J. M. Pong-Wong R. Daetwyler H. D. Calus M. P. (2013). Whole-genome regression and prediction methods applied to plant and animal breeding. Genetics 193, 327–45. 10.1534/genetics.112.143313 22745228 PMC3567727

[B17] FAOSTAT (2020). Food and Agriculture Organization of the United Nations. Available online at: https://www.fao.org/faostat/en/#data/QCL/visualize

[B18] Gebremedhin A. Badenhorst P. Wang J. Shi F. Breen E. Giri K.. (2020). Development and validation of a phenotyping computational workflow to predict the biomass yield of a large perennial ryegrass breeding field trial. Front. Plant Sci. 11, 689. 10.3389/fpls.2020.00689 32547584 PMC7270830

[B19] Gill H. S. Halder J. Zhang J. Brar N. K. Rai T. S. Hall C. (2021). Multi-trait multi-environment genomic prediction of agronomic traits in advanced breeding lines of winter wheat. Front. Plant Sci. 12:709545. 10.3389/fpls.2021.709545 34490011 PMC8416538

[B20] Gilmour A. R. Gogel B. J. Cullis B. R. Welham S. J. Thompson R. (2015). ASReml User Guide Release 4, 1. Functional Specification. Hemel Hempstead: VSN International Ltd.

[B21] Goddard M (2009). Genomic selection: prediction of accuracy and maximisation of long term response. Genetica 136, 245–57. 10.1007/s10709-008-9308-0 18704696

[B22] Goddard M. E. Hayes B. J. (2007). Genomic selection. J. Anim. Breed. Genet. 124, 323–30. 10.1111/j.1439-0388.2007.00702.x 18076469

[B23] Goddard M. E. Hayes B. J. Meuwissen T. H. (2011). Using the genomic relationship matrix to predict the accuracy of genomic selection. J. Anim. Breed. Genet. 128, 409–21. 10.1111/j.1439-0388.2011.00964.x 22059574

[B24] GRDC (2018). GrowNote-Peas-South-1-Introduction. Available online at: https://grdc.com.au/resources-and-publications/grownotes/crop-agronomy/field-pea-southern-region-grownotes/GrowNote-Peas-South-1-Introduction.pdf

[B25] Han L. Yang G. Dai H. Yang H. Xu B. Feng H. (2019). Fuzzy clustering of maize plant-height patterns using time series of UAV remote-sensing images and variety traits. Front. Plant Sci. 10:926. 10.3389/fpls.2019.00926 31379905 PMC6652214

[B26] Hassan M. A. Yang M. Rasheed A. Yang G. Reynolds M. Xia X. (2019). A rapid monitoring of NDVI across the wheat growth cycle for grain yield prediction using a multi-spectral UAV platform. Plant Sci. 282, 95–103. 10.1016/j.plantsci.2018.10.022 31003615

[B27] Hawthorne W. Day T. Pritchard I. Musharaf A. Sykes J. Armstrong E. (2003) Pea Industry History Calender of Events: Breeding. Filed Pea Focus. Wooroloo, WA: Pulse Australia.

[B28] Hayes B. J. Panozzo J. Walker C. K. Choy A. L. Kant S. Wong D. (2017). Accelerating wheat breeding for end-use quality with multi-trait genomic predictions incorporating near infrared and nuclear magnetic resonance-derived phenotypes. Theor. Appl. Genet. 130, 2505–2519. 10.1007/s00122-017-2972-7 28840266

[B29] Holland J. B. Nyquist W. E. Cervantes-Martínez C. T. (2002). “Estimating and interpreting heritability for plant breeding: an update.” *Plant Breeding Reviews*, Vol. 22, ed J. Janick (John Wiley), 9–112. 10.1002/9780470650202.ch2

[B30] Hollaway G. J. Bretag T. W. Price T. V. (2007). The epidemiology and management of bacterial blight (*Pseudomonas syringae* pv. *pisi*) of field pea (*Pisum sativum*) in Australia: A review. Aust. J. Agric. Res. 58, 1086–1099. 10.1071/AR06384

[B31] Huang S. Tang L. Hupy J. P. Wang Y. Shao G. (2020). A commentary review on the use of normalized difference vegetation index (NDVI) in the era of popular remote sensing. J. For. Res. 32, 1–6. 10.1007/s11676-020-01155-1

[B32] Jia Y. Jannink J. L. (2012). Multiple-trait genomic selection methods increase genetic value prediction accuracy. Genetics 192, 1513–22. 10.1534/genetics.112.144246 23086217 PMC3512156

[B33] Khansefid M. Pryce J. E. Bolormaa S. Miller S. P. Wang Z. Li C. (2014). Estimation of genomic breeding values for residual feed intake in a multibreed cattle population. J. Anim. Sci. 92, 3270–83. 10.2527/jas.2014-7375 25074450

[B34] Kreplak J. Madoui M. A. Capal P. Novak P. Labadie K. Aubert G. (2019). A reference genome for pea provides insight into legume genome evolution. Nat. Genet. 51, 1411–1422. 10.1038/s41588-019-0480-1 31477930

[B35] Kumar S. Molloy C. Munoz P. Daetwyler H. Chagne D. Volz R. (2015). Genome-enabled estimates of additive and nonadditive genetic variances and prediction of apple phenotypes across environments. G3 5, 2711–8. 10.1534/g3.115.021105 26497141 PMC4683643

[B36] Lejeune-Henaut I. Hanocq E. Bethencourt L. Fontaine V. Delbreil B. Morin J. (2008). The flowering locus Hr colocalizes with a major QTL affecting winter frost tolerance in *Pisum sativum* L. Theor. Appl. Genet. 116, 1105–16. 10.1007/s00122-008-0739-x 18347775

[B37] Leonforte A. Forster J. W. Redden R. J. Nicolas M. E. Salisbury P. A. (2013). Sources of high tolerance to salinity in pea (*Pisum sativum* L.). Euphytica 189, 203–216. 10.1007/s10681-012-0771-426579164

[B38] Malmberg M. M. Pembleton L. W. Baillie R. C. Drayton M. C. Sudheesh S. Kaur S. (2018). Genotyping-by-sequencing through transcriptomics: implementation in a range of crop species with varying reproductive habits and ploidy levels. Plant Biotechnol. J. 16, 877–889. 10.1111/pbi.12835 28913899 PMC5866951

[B39] Meuwissen T. H. Hayes B. J. Goddard M. E. (2001). Prediction of total genetic value using genome-wide dense marker maps. Genetics 157, 1819–29. 10.1093/genetics/157.4.1819 11290733 PMC1461589

[B40] Oakey H. Verbyla A. Pitchford W. Cullis B. Kuchel H. (2006). Joint modeling of additive and non-additive genetic line effects in single field trials. Theor. Appl. Genet. 113, 809–19. 10.1007/s00122-006-0333-z 16896718

[B41] Pandey A. K. Rubiales D. Wang Y. Fang P. Sun T. Liu N. (2021). Omics resources and omics-enabled approaches for achieving high productivity and improved quality in pea (*Pisum sativum* L.). Theor. Appl. Genet. 134, 755–776. 10.1007/s00122-020-03751-5 33433637

[B42] Piaskowski J. Hardner C. Cai L. Zhao Y. Iezzoni A. Peace C. (2018). Genomic heritability estimates in sweet cherry reveal non-additive genetic variance is relevant for industry-prioritized traits. BMC Genet. 19:23. 10.1186/s12863-018-0609-8 29636022 PMC5894190

[B43] Powers S. E. Thavarajah D. (2019). Checking agriculture's pulse: field pea (*Pisum sativum* L.), sustainability, and phosphorus use efficiency. Front. Plant Sci. 10:1489. 10.3389/fpls.2019.01489 31803218 PMC6873872

[B44] Pritchard I (2015). Growing Field Pea. West Perth, WA: Department of Primary Industries and Regional Development, Western Australia Government.

[B45] Quiros Vargas J. J. Zhang C. Smitchger J. A. McGee R. J. Sankaran S. (2019). Phenotyping of plant biomass and performance traits using remote sensing techniques in pea (*Pisum sativum*, L.). Sensors, 19, 2031. 10.3390/s19092031 PMC6539180 31052251

[B46] Rai R. Singh A. K. Singh B. D. Joshi A. K. Chand R. Srivastava C. P. (2011). Molecular mapping for resistance to pea rust caused by Uromyces fabae (Pers.) de-Bary. Theor. Appl Genet. 123, 803–13. 10.1007/s00122-011-1628-2 21671067

[B47] Robertsen C. D. Hjortshøj R. L. Janss L. L. (2019). Genomic selection in cereal breeding. Agronomy 9, 95. 10.3390/agronomy9020095

[B48] Rutkoski J. Poland J. Mondal S. Autrique E. Perez L. G. Crossa J. (2016). Canopy temperature and vegetation indices from high-throughput phenotyping improve accuracy of pedigree and genomic selection for grain yield in wheat. G3 6, 2799–808. 10.1534/g3.116.032888 27402362 PMC5015937

[B49] Sadras V. O. Lake L. Chenu K. McMurray L. S. Leonforte A. (2012). Water and thermal regimes for field pea in Australia and their implications for breeding. Crop Pasture Sci. 63, 33–44. 10.1071/CP11321

[B50] Singh A. K. Srivastava C. P. (2015). Effect of plant types on grain yield and lodging resistance in pea (*Pisum sativum*L.). Indian J. Genet. Plant Breed. 75, 69–74. 10.5958/0975-6906.2015.00008.5

[B51] Smýkal P. Aubert G. Burstin J. Coyne C. J. Ellis N. T. H. Flavell A. J. (2012). Pea (*Pisum sativum* L.) in the genomic era. Agronomy 2, 74–115. 10.3390/agronomy202007433433637

[B52] Sun J. Rutkoski J. E. Poland J. A. Crossa J. Jannink J. L. Sorrells M. E. (2017). Multitrait, random regression, or simple repeatability model in high-throughput phenotyping data improve genomic prediction for wheat grain yield. Plant Genome 10, plantgenome2016.11.0111. 10.3835/plantgenome2016.11.0111 28724067

[B53] Tayeh N. Aluome C. Falque M. Jacquin F. Klein A. Chauveau A. (2015a). Development of two major resources for pea genomics: the GenoPea 13.2K SNP Array and a high-density, high-resolution consensus genetic map. Plant J. 84, 1257–73. 10.1111/tpj.13070 26590015

[B54] Tayeh N. Aubert G. Pilet-Nayel M. L. Lejeune-Henaut I. Warkentin T. D. Burstin J. (2015b). Genomic tools in pea breeding programs: status and perspectives. Front. Plant Sci. 6:1037. 10.3389/fpls.2015.01037 26640470 PMC4661580

[B55] Van der Auwera G. A. Carneiro M. O. Hartl C. Poplin R. Del Angel G. Levy-Moonshine A. (2013). From FastQ data to high confidence variant calls: the genome analysis toolkit best practices pipeline. Curr. Protoc. Bioinf. 43, 11.10.1–11.10.33. 10.1002/0471250953.bi1110s43 25431634 PMC4243306

[B56] VanRaden P. M (2008). Efficient methods to compute genomic predictions. J. Dairy Sci. 91, 4414–23. 10.3168/jds.2007-0980 18946147

[B57] Velazco J. G. Jordan D. R. Mace E. S. Hunt C. H. Malosetti M. van Eeuwijk F. A. (2019). Genomic prediction of grain yield and drought-adaptation capacity in sorghum is enhanced by multi-trait analysis. Front. Plant Sci. 10:997. 10.3389/fpls.2019.00997 31417601 PMC6685296

[B58] Wang X. Li L. Yang Z. Zheng X. Yu S. Xu C. (2017). Predicting rice hybrid performance using univariate and multivariate GBLUP models based on North Carolina mating design II. Heredity 118, 302–310. 10.1038/hdy.2016.87 27649618 PMC5315526

[B59] Wang X. Xu Y. Hu Z. Xu C. (2018). Genomic selection methods for crop improvement: current status and prospects. Crop J. 6, 330–340. 10.1016/j.cj.2018.03.001

[B60] Yang Z. Shao Y. Li K. Liu Q. Liu L. Brisco B. (2017). An improved scheme for rice phenology estimation based on time-series multispectral HJ-1A/B and polarimetric RADARSAT-2 data. Remote Sens. Environ. 195, 184–201. 10.1016/j.rse.2017.04.016

[B61] Zhang C. Chen W. Sankaran S. (2019). High-throughput field phenotyping of Ascochyta blight disease severity in chickpea. Crop Prot. 125, 104885. 10.1016/j.cropro.2019.104885

[B62] Zhang C. McGee R. J. Vandemark G. J. Sankaran S. (2021). Crop performance evaluation of chickpea and dry pea breeding lines across seasons and locations using phenomics data. Front. Plant Sci. 12:640259. 10.3389/fpls.2021.640259 33719318 PMC7947363

[B63] Zhao H. Li Y. Petkowski J. Kant S. Hayden M. J. Daetwyler H. D. (2021). Genomic prediction and genomic heritability of grain yield and its related traits in a safflower genebank collection. Plant Genome 14, e20064. 10.1002/tpg2.20064 33140563 PMC12807229

[B64] Zohary D (1999). Monophyletic vs. polyphyletic origin of the crops on which agriculture was founded in the Near East. Genet. Resour. Crop Evol. 46, 133–142. 10.1023/A:1008692912820

