# Supplementary material for: Combining NDVI and Bacterial Blight Score to Predict Grain Yield in Field Pea
Source: Front Plant Sci. 2022 Jun 28;13:923381. doi: 10.3389/fpls.2022.923381 (PMC9274273; doi:10.3389/fpls.2022.923381)

Supplementary Figure S1. Rainfall, temperature, and frost hours during 2018 filed pea growing season at the trial site- Horsham.

| 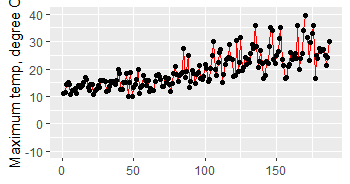 |
| --- |
| 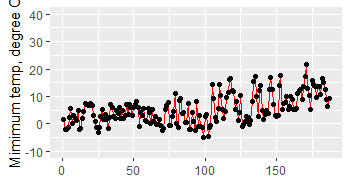 |
| 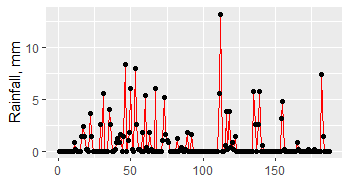  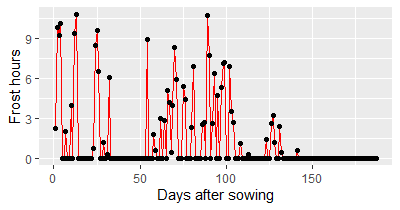 |
|  |

Supplementary Figure S2: Heatmap of the genomic relationship matrix (**G**) of field pea breeding program with total 1453 lines. The color within the GRM indicated the relatedness between breeding lines (high relationships are shown in green, and low relationships are shown in blue).


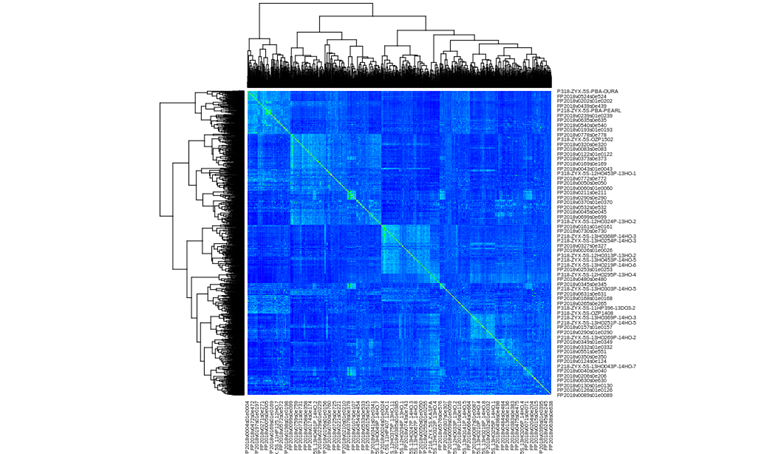


Supplementary Figure S3: Heatmap of the genomic relationship matrix (G) for field pea breeding stages S0, S1, S2, and S3, respectively. The color within the GRM indicated the relatedness between breeding lines (high relationships are shown in green, and low relationships are shown in blue).


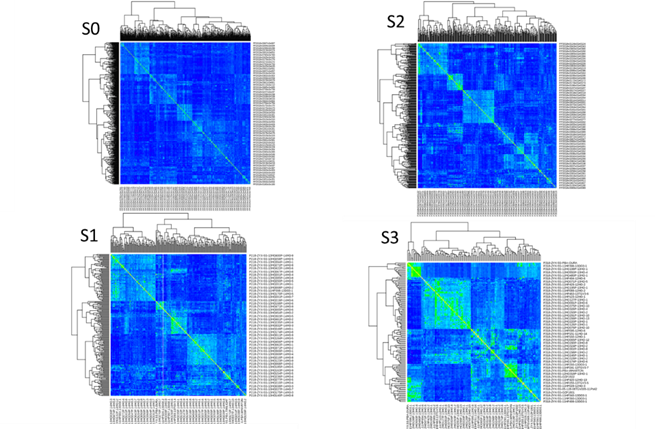


Supplementary Figure S4. The percentage of variance was explained by the first ten principal components (PCs), and the first three PCs plotted against each other for the genomic relationship matrix (G) using all field pea breeding stages.


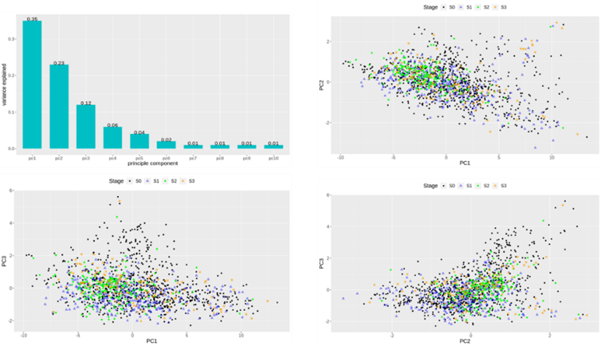

Supplement: Supplementary file 1 [file Data_Sheet_1.docx]
